# Prevalence and factors associated with type 2 diabetes mellitus and hypertension among the hill tribe elderly populations in northern Thailand

**DOI:** 10.1186/s12889-018-5607-2

**Published:** 2018-06-05

**Authors:** Tawatchai Apidechkul

**Affiliations:** 1 Center of Excellence for the Hill tribe Health Research, Mae Fah Luang University, Chiang Rai, Thailand; 2 School of Health Science, Mae Fah Luang University, Chiang Rai, Thailand

**Keywords:** Type 2 diabetes mellitus, Hypertension, Hill tribe, Elderly, Thailand

## Abstract

**Background:**

Type 2 diabetes mellitus (T2DM) and hypertension (HT) are major noncommunicable health problems in both developing and developed countries, including Thailand. Each year, a large amount of money is budgeted for treatment and care. Hill tribe people are a marginalized population in Thailand, and members of its elderly population are vulnerable to health problems due to language barriers, lifestyles, and daily dietary intake.

**Methods:**

An analytic cross-sectional study was conducted to estimate the prevalence of T2DM and HT and to assess the factors associated with T2DM and HT. The study populations were hill tribe elderly adults aged ≥  60 years living in Chiang Rai Province, Thailand. A simple random method was used to select the targeted hill tribe villages and participants into the study. A validated questionnaire, physical examination form, and 5-mL blood specimen were used as research instruments. Fasting plasma glucose and blood pressure were examined and used as outcome measurements. Chi-square tests and logistic regression were used for detecting the associations between variables at the significance level alpha=0.05.

**Results:**

In total, 793 participants participated in the study; 49.6% were male, and 51.7% were aged 60-69 years. A total of 71.5% were Buddhist, 93.8% were uneducated, 62.9% were unemployed, and 89 % earned an income of < 5,000 baht/month. The overall prevalence of T2DM and HT was 16.8% and 45.5%, respectively. Approximately 9.0% individuals had comorbidity of T2DM and HT. Members of the Lahu, Yao, Karen, and Lisu tribes had a greater odds of developing T2DM than did those of the Akha tribe. Being overweight, having a parental history of T2DM, and having high cholesterol were associated with T2DM development. In contrast, those who engaged in highly physical activities and exercise had lower odds of developing T2DM than did those who did not. Regarding HT, being female, having a high dietary salt intake, being overweight, and having a parental history of HT were associated with HT development among the hill tribe elderly populations.

**Conclusions:**

The prevalence of T2DH and HT among the hill tribe elderly populations is higher than that among the general Thai population. Public health interventions should focus on encouraging physical activity and reducing personal weight, dietary salt intake, and greasy food consumption among the hill tribe elderly.

**Electronic supplementary material:**

The online version of this article (10.1186/s12889-018-5607-2) contains supplementary material, which is available to authorized users.

## Background

Type 2 diabetes mellitus (T2DM) and hypertension (HT) are common noncommunicable diseases among elderly adults aged ≥ 60 years in both developing and developed countries [1]. The prevalence of T2DM and HT varies according to age, sex, and race [2, 3]. There are different factors associated with T2DM and HT in different populations, particularly among those with different lifestyles and cultures [3, 4]. Older populations are the most vulnerable to the development of T2DM and HT [5, 6]. T2DM and HT have become major causes of morbidity and mortality of elderly populations in all countries [7, 8]. The impact of T2DM and HT is not limited to physical and mental consequences; rather, it also affects family and national economics [9]. Health professionals in health care institutes must manage the maintenance of plasma glucose levels among T2DM patients and blood pressure among HT patients using different regiments of drugs for their entire lives. With these demands, there are required numbers of health professionals and large amount of financial input needed to operate the treatment and care system each year. Patients need to frequently attend a clinic to meet and receive care from a doctor. Otherwise, many complications could possibly develop, resulting in intensive and complicated methods of treatment and care.

In 2014, the WHO estimated that 422 million people worldwide were suffering from T2DM, which accounted for 8.5% of the prevalence among people over 18 years old. The prevalence is increasing among people aged > 30 years old, particularly in low- and middle-income countries. People aged ≥  60 years old are also commonly defined as a vulnerable population for T2DM [2]. Commonly, T2DM is a disease that progresses slowly from its onset, and it may be diagnosed several years later. T2DM is a major cause of other health problems, such as blindness, kidney failure, heart attacks, stroke, and lower limb amputation. The WHO also reported that 1.6 million deaths were directly caused by diabetes, and almost half of all deaths attributable to high blood glucose occurred before the age of 70 years [2]. This finding reflects the need to regularly investigate those vulnerable to an early diagnosis and determine ways of obtaining a better prognosis. In 2016, the total T2DM prevalence among the Thai population was 9.6%: 9.1% in males and 10.1% in females. The total number of deaths caused by T2DM was 20,570 cases; in the 30-69 year age group, the number of deaths was 8,120 cases (3,610 males, 4,510 females) and in the ≥  70 years age group, the number of deaths was 12,450 cases (4,760 males, 7,690 females). Moreover, total number of deaths attributable to high blood glucose was 35,640 cases; in the 30-69 year age group, the number of deaths was 13,810 cases (7,220 males, 6,590 females) and in the ≥  70 years age group, the number of deaths was 21,830 cases (9,430 males, 12,400 females) [10]. The average cost of each T2DM case in attending hospital services per year was 598US$ for an independent case and 2,700US$ for a disabled case. Therefore, Thailand spends a large amount of money on the health care system annually [11].

High blood pressure is a key risk factor for many diseases, including heart attack and stroke. In 2017, WHO estimated that more than one billion people had HT caused 12.8% of all deaths and accounted for 57 million disability-adjusted life years (DALYs) or a total of 3.7% DALYs every year [12]. Thailand reported that 29.0% of adult Thais had HT, and only 37.0% for those people who had been diagnosed had their blood pressure under control in 2017 [13]. The number of resistant HT patients in all health institutes in the entire country has increased from 3,946,902 cases in 2013 to 5,584,007 cases in 2017 [13]. The statistics represent the full picture of the situation in Thailand, but there is no information available on any specific subgroup of populations, such as the hill tribe population.

The hill tribe people are those who have migrated from the southern region of China to Thailand in the past century [14]. They are divided into six different main groups: Akha, Lahu, Karen, Hmong, Yao, and Lisu [15]. Approximately 2.5 million of the hill tribe people were living in Thailand in 2017 [16]. They have their own culture, language and lifestyles, particularly in daily cooking. Some tribes use a high volume of oil for cooking, whereas other tribes use a high volume of salt for their daily food [14, 17]. Most of them have similar cultural patterns in terms of using alcohol, particularly for religious rituals [18].

In 2017, the hill tribe elderly populations lived according to their own traditional lifestyle and living environment. They consumed drinks and foods prepared traditionally. Individual health care was mainly based on their local healing patterns. With the problems of distance, language and discrimination, their access to the Thai health care system was poor [19]. Therefore, access to modern medical care is not common, especially for those who live very far from the city. Ultimately, the findings of the study could support the development of the health care service system for the hill tribe elderly populations. The findings could also be used for the development of DM and HT prevention and control measures in these populations. Currently, there is no available information about T2DM and HT among these population groups. Therefore, the study aimed to estimate the prevalence and factors associated with DM and HT among the hill tribe elderly populations in northern Thailand.

## Methods

### Study design and participants

#### Study design

A cross-sectional study was conducted to gather information from the selected subjects.

#### Study setting

The study was conducted along 16 districts in Chiang Rai Province, which is located in Thailand.

#### Study population

The study population was comprised of hill tribe elderly adults aged ≥ 60 years old who had lived in the study setting for at least 3 years.

#### Eligible population

Elderly adults with the following characteristics were eligible for the study: a) being classified as a member of the hill tribe by verbal confirmation, b) being ≥ 60 years old, c) living in the study area for 3 years at the date of data collection, and d) having the ability to provide essential information. Those who had been diagnosed with type 1 diabetes mellitus, which requires daily administration of insulin, were excluded from the study.

#### Sample size

The sample size was calculated by Epi-Info version 7.2 (US Centers for Disease Control and Prevention, Atlanta, GA). By setting the alpha error at 0.05, the power at 0.8, the previous prevalence of T2DM among the exposed group at 18.0%, and the prevalence among the unexposed group at 0.07% [20], the sample size was calculated to be a minimum of 705 participants. Increasing the sample size by 10.0% for error resulted in 775 participants required.

Since the sample size was calculated at 775 participants, at least 130 participants were needed in each tribe.

#### Sample selection and preparing the participants

The list of the hill tribe villages in Chiang Rai Province was requested from the Hill Tribe Welfare and Development Center in Chiang Rai [21]. There were 749 hill tribe villages in Chiang Rai, which breakdown into 316 Lahu villages, 243 Akha villages, 63 Yao villages, 56 Hmong villages, 36 Karen villages, and 35 Lisu villages. In 2016, a total of 41,366 hill tribe families lived in Chiang Rai Province.

**Fig. 1:**
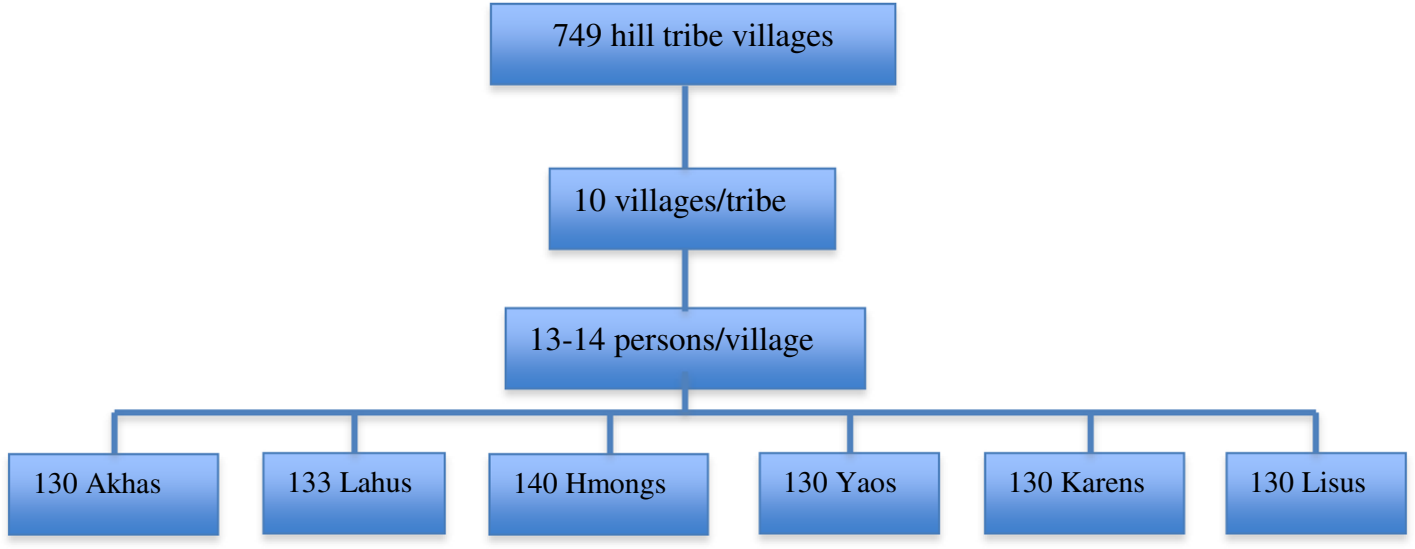
Flowchart of participants’ selection. Sixty hill tribe villages were randomly selected from 749 villages, and 13-14 elderly people in each village were recruited into the study

Permission to access the villages had been granted by the District Government Officer. Sixty hill tribe villages, or 10 villages in each tribe, were selected by a simple random method. A village headman was contacted and informed of all essential information regarding the research objective and its protocol. The list of elderly people who met the inclusion and exclusion criteria in the village was sent to the researcher. A simple random method was used to select 13 individuals in each village, after which they were invited to participate in the study. After informing the village headman about the research objectives and protocols, some tribes collected more than the minimum required sample size: Lahu (an excess of 3 participants) and Hmong (an excess of 10 participants). Those who agreed to join the project were informed of all research processes, including the preparation of NPO (nothing per oral) for at least 8 hours for the blood specimen collection on the next day (Fig. 1).

Six research assistants fluent in Thai and in one of the six hill tribal languages were recruited. Selected research assistants were trained in procedures, and the required documents were completed three days before working in the field. Most of the hill tribe elderly populations do not speak Thai. Therefore, there was a need to obtain complete information using the research assistants. Recruiting young adults to help as research assistants was possible because hill tribe community members younger than 25 years old had already completed secondary level education in Thai schools.

### Measurement

#### Research instruments

A questionnaire, physical examination form, a manual sphygmomanometer, and a 5-mL blood specimen served as research instruments. A questionnaire was developed from the review literature. After completion of the first draft, the validity was detected by the item-objective congruence (IOC) technique in which three external experts in relevant fields verified the validity. Questions with scores less than 0.5 were excluded, those with scores 0.5-0.69 were revised, and those with scores greater than 0.7 were defined as acceptable to use. The questionnaire was also tested for reliability by pilot testing it in 15 similar participants in the Ban San Ti Suk hill tribe village using the test-retest method. Questions with Cronbach’s alpha ≥ 0.5 were included in the form. Ultimately, there were 28 questions in three parts included in the questionnaire, which presented an overall Cronbach’s alpha of 0.77.

In the first parts, 13 questions were used to collect participants’ general information, such as age, sex, education, and religion. Fifteen questions were included in the second part, including questions about health behaviors such as “Do you smoke?”, “Do you drink alcohol?”, and “Do you use methamphetamine?”. In these questions, the three answer choices were “Yes”, “Ever in the past”, and “No”.

Several questions were asked regarding daily food consumption and exercise, such as “Do you usually eat a salty diet?”, “Do you favor having greasy food?”, and “Do you like to eat sweet food?” Two answer choices were provided for the questions, “Yes” and “No”. However, for exercise practices, the three answer choices were “No”, “Highly active physical work, such as farmer and labor”, and “Yes”.

Questions on medications, history of T2DM and HT, and parental history of T2DM and HT were also included. To confirm the diagnosis, all participants who responded that they had T2DM and HT were asked to present the log-book from a hospital. In Thailand, all DM and HT cases are provided individual log-books to use to collect medical information and make appointments.

The last part consisted of twenty-one items of a physical examination form, which is used at Mae Fah Luang University Hospital. This form resembles a checklist and can include more information if required. A manual sphygmomanometer was used for assessing blood pressure.

#### Variables and measurements

T2DM in the study was identified by the following: a) having no history of a medical diagnosis, such as type 1 diabetes mellitus, at a particularly early age after birth, b) having shown a fasting plasma glucose ≥ 126 mg/dL twice on different days [20].

Blood pressure was assessed twice in all participants with a 15-minute gap between assessments, and in case systolic and/or diastolic blood pressures were greater than 90 mmHg and/or 150 mmHg, respectively, it was assessed again after 15 minutes of rest following the 2^nd^ assessment. Participants with 90 mmHg and/or 140 mmHg of systolic and diastolic blood pressures, respectively, were diagnosed as HT patients [22].

Body mass index (BMI) was classified into three categories, according to the WHO guidelines for Asian populations: underweight (BMI≤18.5), normal weight (BMI=18.51-22.99), and overweight (BMI ≥ 23.00) [23].

A 5-mL blood specimen was collected from a peripheral vein puncture. After blood was drawn, a 3-mL blood specimen was collected and stored in a sodium fluoride tube to detect fasting plasma glucose. Another 2-mL blood specimen was collected and stored in a clot blood clot tube for detecting lipid profiles. Uric acid, cholesterol, and triglycerides were assessed in mg/dL. Participants with uric acid ≥ 7 mg/dL, cholesterol ≥ 200 mg/dL, and triglycerides ≥ 200 mg/dL were defined as a high-level group [24]. Participants with fasting plasma glucose ≥ 126 mg/dL were asked to provide another blood specimen within a week to determine type 2 diabetes stage.

### Procedures

#### Data gathering procedures

After the consent form was obtained, a 5-mL blood specimen was collected. Participants were asked to complete the questionnaire in a private room in the village with the help of the research assistants. A trained physician examined the physical health of all participants in a proper room. A small gift was given to participants after they completed the questionnaire.

### Statistical analysis

Descriptive statistics, such as the means, minimums, maximums, standard deviations, and percentages, were used to explain the general characteristics of the participants. Chi-square tests and logistic regressions were used to detect the associations between variables at the significance level α=0.05. Logistic regression was used to detect the associations between variables in both univariate and multivariate models. The “ENTER” mode was used to select the significant variables in the model. The significance level (alpha) was set at 0.05 in both univariate and multivariate analyses. Variables that were found to be significant in the univariate analysis were retained in the multivariate analysis. In the multivariate model, the most nonsignificant variable was deleted from the model before running the second step. The model was analyzed until all remaining variables were found to be significant at an alpha level of 0.05, and the results were interpreted.

## Results

### Characteristics of participants

In total, 793 participants were recruited into the study. Proportions of participants were mostly equal by sex and among the six tribes. A few people had no Thai identification card (6.1%), with an equal proportion among the tribes. The majority were aged 60-69 years (51.7%), with an average age of 70.1 years (range=60-100, SD=7.57, max=100, and min=60). The majority of the sample practiced Buddhism (71.5%) and had no education (94.8%). A few people lived alone (6.1%), and most participants were married (66.8%). Regarding economic status, 89.2% had an income of ≤ 5,000 baht/month (mean=1,129 baht, SD=1,273), and 84.9% had no debt (Table 1).

**Table 1:** General characteristics of the study participants

Characteristics	Number	Percent
Total	793	100.0
Sex		
Male	393	49.6
Female	400	50.4
Thai ID card		
Yes	745	93.9
No	48	6.1
Tribe		
Akha	130	16.4
Lahu	133	16.8
Hmong	140	17.6
Yao	130	16.4
Karen	130	16.4
Lisu	130	16.4
Age (years)		
60-69	410	51.7
70-79	279	35.2
≥ 80	104	13.1
Religion		
Buddhism	567	71.5
Christianity	225	28.4
Islam	1	0.1
Education		
None	739	93.8
Primary School	41	5.2
High School	8	1.0
Resides with		
Child	559	70.5
Cousin	12	1.5
Spouse	174	21.9
Alone	48	6.1
Marital status		
Single	15	1.9
Married	524	66.8
Divorced	20	2.5
Widow	226	28.8
Number of family member (persons)		
1	40	5.0
2	116	14.6
3-5	301	38.0
6	336	42.4
Occupation		
Unemployed (retired)	499	62.9
Farmer	252	31.8
Merchant	11	1.4
Labor	19	2.4
Other	12	1.5
Monthly family income (baht)		
0	69	8.7
≤5,000	707	89.2
≥5,001	17	2.1
Debt (baht)		
0	673	84.9
≤5,000	14	1.8
5,001-10,000	11	1.4
10,001-50,000	58	7.3
≥50,001	37	4.6

There were no statistical differences in the distribution of participants according to sex and tribe in three different age categories (60-69, 70-79, and ≥ 80 years). A few of the hill tribe elderly adults had the ability to communicate in Thai: 19.5% could speak, 19.5% could understand, 2.0% could read, and 1.6% could write fluently. Males had significantly better Thai communication skills than females in all four domains: speaking, understanding, reading, and writing.

The prevalence of T2DM and HT was 16.8% and 45.5%, respectively. Seventy-five participants had been diagnosed with T2DM before being recruited into the study. Among these participants, 8 (10.6%) had high fasting glucose or were unable to control blood glucose after medication. Fifty-five participants (7.7%) were detected as new T2DM cases (Table 2). However, 18 participants (1.2%) could not draw blood specimens.

**Table 2:** Prevalence of T2DM and HT among the participants

Chracteristics	Number	Percent
Medical history of T2DM		
No	718	90.5
Yes	75	9.5
Effective control of blood glucose by daily medication		
No	8	10.6
Yes	67	89.4
Fasting plasma glucose level among non-DM diagnosed		
Normal	645	89.8
High (T2DM)	55	7.7
(Missing=18, 2.5%)		
^a^Prevalence of T2DM=16.8%		
Medical history of HT		
No	553	69.7
Yes	240	30.3
Effective control of blood pressure by daily medication		
No	91	37.9
Yes	149	62.1
Blood pressure level among non-HT diagnosed		
Normal	432	78.1
High (HT)	121	21.9
^b^Prevalence of HT=45.5%		
Having both T2DM and HT	70	9.0

^a^ The overall prevalence of T2DM among the participants

^b^ The overall prevalence of HT among the participants

Two hundred and forty participants (30.3%) had been diagnosed with HT, among whom 37.9% were unable to control their blood pressure after medication. After those who had no history of HT diagnosis and medication were seen, 121 participants (21.9%) were detected as new HT cases. Finally, 70 cases (9.0%) were determined to have both T2DM and HT: 36 males and 34 females (Table 2).

There was statistical significance in the proportion of participants with T2DM and HT by sex and tribe. Only the participants with T2DM showed a statistically significant difference in proportion (Table 3).

**Table 3:** Comparison of T2DM and HT by participants’ characteristics

Characteristic	T2DM		χ^2^	*p*-value	HT		χ^2^	*p*-value
	Yes (%)	No (%)			Yes (%)	No (%)		
Sex								
Male	66 (17.3)	316 (82.7)	0.13	0.712	164 (41.7)	229 (58.3)	4.52	0.034*
Female	64 (16.3)	329 (83.7)			197 (49.3)	203 (50.7)		
Age (years)								
60-69	75 (18.8)	324 (81.2)	2.49	0.287	173 (42.2)	237 (57.8)	4.25	0.119
70-79	39 (14.3)	234 (85.7)			134 (48.0)	145 (52.0)		
≥80	16 (15.5)	87 (84.5)			54 (51.9)	50 (48.1)		
Tribe								
Akha	11 (8.6)	117 (91.4)	24.48	<0.001*	61 (46.9)	69 (53.1)	26.45	<0.001*
Lahu	26 (19.5)	107 (80.5)			61 (45.9)	72 (54.1)		
Hmong	11 (8.1)	124 (91.9)			42 (30.0)	98 (70.0)		
Yao	26 (21.5)	95 (78.5)			74 (56.9)	56 (43.1)		
Karen	34 (26.4)	95 (73.6)			52 (40.0)	78 (60.0)		
Lisu	22 (17.1)	107 (82.9)			71 (54.6)	59 (45.4)		

*Significance level at α=0.05

Health behaviors among the participants indicated that 19.7% smoked, 14.6% drank alcohol, 44.9% ate uncooked food, 23.8% chewed tobacco, and 10.1% did not exercise regularly. A comparison of health behaviors such as smoking, alcohol use, eating uncooked food, and regular exercise among the tribes showed statistically significant differences (Table 4). Additionally, there were significant sex differences in the following health behaviors: smoking; alcohol use; the consumption of uncooked food, salty food, greasy food, and sweet food; opium use; chewing tobacco; and regular exercise (Table 5).

**Table 4:** Characteristics of health behaviors by tribe

Health behaviors	Tribe														χ^2^	*p*-value
	Total		Akha		Lahu		Hmong		Yao		Karen		Lisu			
	n	%	n	%	n	%	n	%	n	%	n	%	n	%		
Smoking																
No	486	61.3	94	19.3	70	14.4	106	21.8	72	14.8	48	9.9	96	19.8	79.02	< 0.001*
Ever in the past	151	19.0	12	7.9	33	21.9	11	7.3	29	19.2	50	33.1	16	10.6		
Yes	156	19.7	24	15.4	30	19.2	23	14.7	29	18.6	32	20.5	18	11.5		
Alcohol use																
No	538	67.8	99	18.4	92	17.1	109	20.3	88	16.4	77	14.3	73	13.6	43.93	< 0.001*
Ever	139	17.5	13	9.4	29	20.9	14	10.1	17	12.2	25	18.0	41	29.5		
Yes	116	14.6	18	15.5	12	10.3	17	14.7	25	21.6	28	24.1	16	13.8		
Methamphetamine use																
No	776	97.9	124	16.0	132	17.0	137	17.7	126	16.4	128	16.5	129	16.6	12.15	0.275
Ever in the past	2	0.3	0	0.0	0	0.0	0	0.0	1	50.0	1	50.0	0.0	0.0		
Yes	15	1.9	6	40.0	1	6.7	3	20.0	3	20.0	1	6.7	1	6.7		
Opium use																
No	723	91.2	112	15.5	125	17.3	124	17.2	115	15.9	123	17.0	124	17.2	15.77	0.106
Ever in the past	54	6.8	12	22.2	6	11.1	12	22.2	12	22.2	7	13.0	5	9.3		
Yes	16	2.0	6	37.5	2	12.5	4	25.0	3	18.8	0	0.0	1	6.3		
Eating uncooked food																
No	385	48.5	79	20.5	74	19.2	68	17.7	69	17.9	43	11.2	52	13.5	29.65	< 0.001*
Ever in the past	52	6.6	5	9.6	6	11.5	9	17.3	8	15.4	11	21.2	13	25.0		
Yes	356	44.9	46	12.9	53	14.9	63	17.7	53	14.9	76	21.3	65	18.3		
Chewing																
No	604	76.2	70	11.6	108	17.9	135	22.4	128	21.2	100	16.6	63	10.4	159.80	< 0.001*
Yes	189	23.8	60	31.7	25	13.2	5	2.6	2	1.1	30	15.9	67	35.4		
Regular exercise																
No	80	10.1	22	27.5	7	8.8	15	18.8	7	8.8	22	27.5	7	8.8	37.50	< 0.001*
Yes	433	54.6	68	15.7	88	20.3	75	17.3	66	15.2	54	12.5	82	18.9		
Highly active physical work	280	35.3	40	14.3	38	13.6	50	17.9	57	20.4	54	19.3	41	14.6		

*Significance level at α=0.05

**Table 5:** Comparison of health behavior by sex

Health behvaior	Total		Male		Female		χ^2^	*p*-value
	n	%	n	%	n	%		
Smoking								
No	486	61.3	151	31.1	335	68.9	173.52	< 0.001*
Ever in the past	151	19.0	125	82.8	26	17.2		
Yes	156	19.7	117	75.0	39	25.0		
Alcohol use								
No	538	67.8	169	31.4	369	68.6	222.02	< 0.001*
Ever in the past	139	17.5	117	84.2	22	15.8		
Yes	116	14.6	107	92.2	9	7.8		
Consumption of uncooked food								
No	385	48.5	106	27.5	279	72.5	145.24	< 0.001*
Ever in the past	52	6.6	37	71.2	15	28.8		
Yes	356	44.9	250	70.2	106	29.8		
Salty food								
No	282	35.6	106	37.6	176	62.4	25.05	< 0.001*
Yes	511	64.4	287	56.2	224	43.8		
Greasy food								
No	297	37.5	194	65.3	103	34.7	47.12	< 0.001*
Yes	496	62.5	199	40.1	297	59.9		
Sweet food								
No	391	49.3	216	55.2	175	44.8	9.96	0.0016*
Yes	402	50.7	177	44.0	225	56.0		
Opium use								
No	723	91.2	339	46.9	384	53.1	23.95	< 0.001*
Ever in the past	54	6.8	43	79.6	11	20.4		
Yes	16	2.0	11	68.8	5	31.3		
Methamphetamine use								
No	776	97.9	381	49.1	395	50.9	3.69	0.079
Yes	17	2.1	12	70.6	5	29.4		
Chewing								
No	604	76.2	313	51.8	291	48.2	5.19	0.023*
Yes	189	23.8	80	42.3	109	57.7		
Regular exercise								
No	433	54.6	184	42.5	249	57.5	26.05	< 0.001*
Highly active physical work	280	35.3	173	61.8	107	38.2		
Yes	80	10.1	36	45.0	44	55.0		

*Significance level at α=0.05

Most participants had moderate levels of health-related knowledge, attitudes, and practices. Only the distribution of attitudes by tribe showed statistical significance (Table 6).

**Table 6:** Comparison on knowledge, attitudes, and practices regarding health among tribes

KAP			Tribe												χ^2^	*p*-value
	Total		Akha		Lahu		Hmong		Yao		Karen		Lisu			
	n	%	n	%	n	%	n	%	n	%	n	%	n	%		
Total	377	100.0	60	15.9	76	20.2	46	12.2	70	18.6	73	19.4	52	13.8		
Knowledge																
Low	61	16.2	15	24.6	13	21.3	10	16.4	8	13.1	5	8.2	10	16.4	15.07	0.129
Moderate	167	44.3	24	14.4	33	19.8	21	12.6	38	22.8	31	18.6	20	12.0		
High	149	39.5	21	14.1	30	20.1	15	10.1	24	16.1	37	24.8	22	14.8		
Attitude																
Low	53	14.1	12	22.6	5	9.4	14	26.4	14	26.4	4	7.5	5	9.4	38.04	< 0.001*
Moderate	250	66.3	44	17.6	55	22.0	25	10.0	42	16.8	44	17.6	40	16.0		
High	74	19.6	4	5.4	16	21.6	7	9.5	14	18.9	25	33.8	8	10.8		
Practice																
Low	47	12.5	3	6.4	8	17.0	10	21.3	9	19.1	8	17.0	9	19.1	10.51	0.397
Moderate	267	70.8	44	16.5	56	21.0	27	10.1	49	18.4	54	20.2	37	13.9		
High	63	16.7	13	20.6	12	19.0	9	14.3	12	19.0	11	17.5	6	9.5		

*Significance level at α=0.05

With regard to the physical health and medical history among the participants, 45.0% were overweight, 6.8% were disabled persons, 15.0% had sleeping problems, 9.7% had cataracts, 28.7% had hearing problems, and 43.3% had tooth problems (Table 7).

**Table 7:** Physical examination and medical history

Item	Total		Male		Female		χ^2^	*p*-value
	n	%	n	%	n	%		
BMI								
Underweight	116	14.6	62	53.4	54	46.6	3.98	0.137
Normal	320	40.4	168	52.5	152	47.5		
Overweight	357	45.0	163	45.7	194	54.3		
Disabled								
No	739	93.2	362	49.0	377	51.0	1.42	0.232
Yes	54	6.8	31	57.4	23	42.6		
Heart disease								
No	724	96.1	337	46.5	387	53.5	0.37	0.538
Yes	29	3.9	16	55.2	13	44.8		
History of TB diagnosis								
No	757	95.5	369	48.7	388	51.3	4.41	0.036*
Yes	36	4.5	24	66.7	12	33.3		
Sleeping problem								
No	674	85.0	356	52.8	318	47.2	19.09	< 0.001*
Yes	119	15.0	37	31.1	82	68.9		
Eye								
Normal	663	83.6	328	49.5	335	50.5	0.99	0.804
Cataract	77	9.7	36	46.8	41	53.2		
Pterygium	50	6.3	27	54.0	23	46.0		
History of glaucoma	3	0.4	2	66.7	1	33.3		
Tooth problem								
No	450	56.7	234	52.0	216	48.0	2.48	0.115
Yes	343	43.3	159	46.4	184	53.6		
Headache								
No	557	72.1	302	54.2	275	49.4	6.55	0.010*
Yes	216	27.9	91	42.1	125	57.9		
Dizziness								
No	556	70.1	294	52.9	262	47.1	8.19	0.004*
Yes	237	29.9	99	41.8	138	58.2		
Peptic ulcer								
No	527	66.5	278	52.8	249	47.2	6.40	0.011*
Yes	266	33.5	115	43.2	151	56.8		
Anorexia								
No	707	89.2	371	52.5	336	47.5	22.18	< 0.001*
Yes	86	10.8	22	25.6	64	74.4		
History of injury								
No	713	89.9	349	48.9	364	51.1	1.05	0.305
Yes	80	10.1	44	55.0	36	45.0		
History of hospital admission								
No	310	39.1	143	46.1	167	53.9	2.39	0.122
Yes	483	60.9	250	51.8	233	48.2		
Parental history of DM								
No	515	64.9	262	50.9	253	49.1	1.01	0.313
Yes	278	35.1	131	47.1	147	52.9		
Parental history of HT								
No	375	47.3	190	50.7	185	49.3	0.34	0.554
Yes	418	52.7	203	48.6	215	51.4		

*Significance level at α=0.05

There were statistically significant differences in the quality of uric acid and cholesterol according to sex, age category, and tribe. A greater proportion of males, individuals in higher age categories, and Lahu and Lisu tribe members had high uric acid levels than did females, those in younger age categories, and members of other tribes. Only age category and tribe showed significant differences on the level of triglycerides; a greater proportion of those in lower age categories had high cholesterol than those in higher age categories. A greater proportion of members of the Lahu and Akha tribes were in the high cholesterol group compared to those in the remaining tribes (Table 8).

**Table 8:** Classification of participants’ characteristics by biomarkers

Factors	Uric acid		χ^2^	*p*-value	Cholesterol		χ^2^	*p*-value	Triglyceride		χ^2^	*p*-value
	Normal n (%)	High n (%)			Normal n (%)	High n (%)			Normal n (%)	High n (%)		
Sex												
Male	246 (64.4)	136 (35.6)	38.63	<0.001*	286 (74.9)	96 (25.1)	14.28	<0.001*	309 (80.9)	73 (19.1)	2.44	0.118
Female	329 (83.9)	63 (16.1)			244 (67.4)	148 (32.6)			299 (76.3)	93 (23.7)		
Age (years)												
60-69	311 (77.9)	88 (22.1)	6.04	0.049*	261 (65.4)	138 (34.6)	4.45	0.108*	303 (75.9)	96 (24.1)	6.58	0.037*
70-79	197 (71.1)	80 (28.9)			195 (78.9)	82 (21.1)			219 (79.1)	58 (20.9)		
≥ 80	67 (68.4)	31 (31.6)			74 (78.7)	24 (21.3)			86 (87.8)	12 (12.2)		
Tribe												
Akha	101 (78.3)	28 (21.7)	20.19	0.018*	95 (73.6)	34 (26.4)	17.05	0.004*	99 (76.7)	30 (23.3)	8.86	0.114
Lahu	113 (85.0)	20 (15.0)			100 (75.2)	33 (24.8)			96 (72.2)	37 (27.8)		
Hmong	81 (64.3)	45 (35.7)			93 (73.8)	33 (26.2)			100 (79.4)	26 (20.6)		
Yao	96 (74.4)	33 (25.6)			82 (90.0)	47 (9.1)			98 (75.9)	31 (24.1)		
Karen	99 (76.7)	30 (23.3)			72 (55.8)	57 (44.2)			111 (86.0)	18 (14.0)		
Lisu	85 (66.4)	43 (33.6)			88 (68.8)	40 (31.2)			104 (81.3)	24 (18.7)		

*Significance level at α=0.05

In the multivariate model, five factors were associated with T2DM: tribe, exercise, BMI, parental history of T2DM, and triglycerides. The Lahu, Yao, Karen, and Lisu tribes had greater odds of developing T2DM than the Akha tribe, with OR_adj_=2.89 (95%CI=1.32-6.33), OR_adj_=3.47 (95%CI=1.58-7.62), OR_adj_=5.03 (95%CI=2.35-10.78), and OR_adj_=2.73 (95%CI=1.22-6.07) respectively. Those who were overweight had greater odds of developing T2DM than those with normal weight, with OR_adj_=2.08 (95%CI=1.32-3.27). Those who had a parental history of T2DM had greater odds of developing T2DM than those who did not, with OR_adj_=1.55 (95%CI=1.17-2.10). Those with high cholesterol had greater odds of developing T2DM than those with low cholesterol, with OR_adj_=1.73 (95%CI=1.10-2.73). Those who engaged in high levels of physical activity and exercise had lower odds of developing T2DM than those who did not, with OR_adj_=0.48 (95%CI=0.25-0.91) and OR_adj_=0.45 (95%CI=0.24-0.83), respectively (Table 9).

**Table 9:** Factors associated with T2DM in univariate and multivariate analyses (*n* = 775)**

Factors	T2DM				OR	95%CI	*p*-value	OR_adj_	95%CI	*p*-value
	Yes		No							
	n	%	n	%						
Sex										
Mal	66	17.3	316	82.7	1.00					
Female	64	16.3	329	83.7	0.93	1.02 -2.02	0.712			
Tribe										
Akha	11	8.6	117	91.4	1.00			1.00		
Lahu	26	19.5	107	80.5	2.58	1.37-4.85	0.013*	2.89	1.32-6.33	0.008*
Hmong	11	8.1	124	91.9	0.94	0.45-1.96	0.896	0.91	0.35-2.31	0.845
Yao	26	21.5	95	78.5	2.91	1.54-5.48	0.006*	3.47	1.58-7.62	0.002*
Karen	34	26.4	95	73.6	3.80	2.06-7.03	< 0.001*	5.03	2.35-10.78	< 0.001*
Lisu	22	17.1	107	82.9	2.18	1.14-4.13	0.046*	2.73	1.22-6.07	0.014*
Age (year)										
60-69	75	18.8	324	81.2	1.00					
70-79	39	14.3	234	85.7	0.72	0.50-1.02	0.127			
≥ 80	16	15.5	87	84.5	0.79	0.48-1.30	0.444			
Smoking										
No	78	16.4	398	83.6	1.00					
Ever in the past	34	23.1	113	76.9	1.53	1.04-2.24	0.064*			
Yes	18	11.8	134	88.2	0.68	0.43-1.08	0.177			
Alcohol use										
No	79	15.0	447	85.0	1.00					
Ever in the past	26	19.3	109	80.7	1.35	0.89-2.03	0.230			
Yes	25	21.9	89	78.1	1.58	1.04 -2.42	0.072*			
Salty food										
No	151	53.5	131	46.5	1.00					
Yes	266	52.1	245	47.9	0.94	0.70-1.26	0.687			
Greasy food										
No	155	52.2	142	47.8	1.00					
Yes	258	52.0	238	48.0	0.99	0.74-1.32	0.962			
Sweet food										
No	202	51.7	189	48.3	1.00					
Yes	184	45.8	218	54.2	0.78	0.59-1.04	0.097			
Exercise										
No	21	26.6	58	73.4	1.00			1.00		
Highly active physical work	45	16.7	225	83.3	0.55	0.33- 0.90	0.050*	0.48	0.25-0.91	0.024*
Yes	64	15.0	362	85.0	0.48	0.30- 0.78	0.013*	0.45	0.24-0.83	0.011*
BMI										
Normal	39	12.6	271	87.4	1.00			1.00		
Underweight	13	11.4	101	88.6	0.89	0.51-1.56	0.743	0.90	0.45-1.80	0.773
Overweight	78	22.2	273	77.8	1.98	1.39- 2.82	0.001*	2.08	1.32-3.27	0.001*
Parental history of DM										
No	217	42.1	298	57.9	1.00			1.00		
Yes	149	53.6	129	46.4	1.58	1.18-2.12	0.002*	1.55	1.17-2.10	0.001*
Hypertension										
No	70	12.9	282	80.1	1.00					
Yes	60	14.2	363	85.8	1.50	1.02- 2.19	0.035*			
Headache										
No	95	16.9	467	83.1	1.00					
Yes	35	16.4	178	83.6	0.96	0.67-1.38	0.875			
Dizziness										
No	86	15.9	456	84.1	1.00					
Yes	44	18.9	189	81.1	1.23	0.88-1.72	0.303			
Cholesterol										
Normal	90	17.3	430	82.7	1.00					
High	38	16.1	198	83.9	0.91	0.64-1.29	0.682			
Triglyceride										
Normal	88	14.9	504	85.1	1.00			1.00		
High	40	24.4	124	75.6	1.84	1.29-2.63	0.004*	1.73	1.10-2.73	0.017*

*Significance level at α=0.05 **18 participants could not provide blood specimens

Four factors were found to be associated with HT after controlling for all possible confounding variables: sex, dietary salt intake, BMI, and parental history of HT. Females had greater odds of developing HT than males, with OR_adj_=1.29 (95%CI=1.01-1.68). Those who had dietary salt intake had greater odds of developing HT than those who did not, with OR_adj_=1.48 (95%CI=1.14-2.00). Those who were overweight had greater odds of developing HT than those with normal weight, with OR_adj_=1.37 (95%CI=1.01-1.90), and those who had a parental history of HT had greater odds of developing HT than those who did not, with OR_adj_=3.38 (95%CI=2.81-4.48) (Table 10).

**Table 10:** Factors associated with HT in univariate and multivariate analyses

Factors	HT				OR	95%CI	*p*-value	OR_Adj_	95%CI	*p*-value
	Yes		No							
	n	%	n	%						
Sex										
Male	164	41.7	229	58.3	1.00			1.00		
Female	197	49.3	203	50.7	1.35	1.02-1.79	0.034*	1.29	1.01-1.68	0.031*
Tribe										
Akha	61	46.9	69	53.1	1.00					
Lahu	61	45.9	72	54.1	0.95	0.59-1.55	0.863			
Hmong	42	30.0	98	70.0	0.48	0.29-0.79	0.004*			
Yao	74	56.9	56	43.1	1.49	0.91-2.43	0.107			
Karen	52	40.0	78	60.0	0.75	0.46-1.23	0.261			
Lisu	71	54.6	59	45.4	1.36	0.83-2.21	0.215			
Age (years)										
60-69	173	42.2	237	57.8	1.00					
70-79	134	48.0	145	52.0	1.26	0.93-1.71	0.131			
≥80	54	51.9	50	48.1	1.48	0.96-2.27	0.075			
Smoking										
No	233	47.9	253	52.1	1.00					
Ever in the past	65	43.0	86	57.0	0.82	0.56-1.18	0.293			
Yes	63	40.4	93	59.6	0.73	0.51-1.06	0.100			
Alcohol use										
No	249	46.3	289	53.7	1.00					
Ever in the past	64	46.0	75	54.0	0.99	0.68-1.44	0.960			
Yes	48	41.4	68	58.6	0.81	0.54-1.23	0.337			
Salty food										
No	138	48.9	144	51.1	1.00			1.00		
Yes	307	60.1	204	39.9	1.57	1.17-2.01	0.002*	1.48	1.14-2.00	0.001*
Greasy food										
No	136	45.8	161	54.2	1.00					
Yes	241	48.6	255	51.4	1.11	0.83-1.49	0.582			
Sweet food										
No	202	51.7	189	48.3	1.00					
Yes	197	49.0	205	51.0	0.89	0.68-1.18	0.454			
Regular Exercise										
Yes	36	45.0	44	55.0	1.00					
Highly active physical work	113	40.5	166	59.5	0.83	0.50-1.37	0.472			
No	212	48.8	222	51.2	1.16	0.72-1.88	0.527			
BMI										
Normal	112	35.0	208	65.0	1.00			1.00		
Underweight	42	36.2	74	63.8	1.05	0.67-1.64	0.816	2.56	0.70 – 1.70	0.696
Overweight	207	58.0	150	42.0	2.56	1.87- 3.49	< 0.001*	1.37	1.01 – 1.90	< 0.001*
Parental history of HT										
No	155	41.3	220	58.7	1.00			1.00		
Yes	302	72.2	116	27.8	3.69	2.74-4.97	< 0.001*	3.38	2.81-4.48	< 0.001*
Diabetes mellitus										
No	282	43.7	363	56.3	1.00					
Yes	70	53.8	60	46.2	1.50	1.02- 2.19	0.035*			
Headache										
No	249	43.2	328	56.8	1.00					
Yes	112	51.9	104	48.1	1.41	1.03-1.94	0.029*			
Dizziness										
No	238	42.8	318	57.2	1.00					
Yes	123	51.9	114	48.1	1.44	1.06-1.95	0.019*			
Cholesterol										
Normal	238	44.9	292	55.1	1.00					
High	115	47.1	129	52.9	1.09	0.80-1.48	0.564			
Triglyceride										
Normal	262	43.1	346	56.9	1.00					
High	91	54.8	75	45.2	1.60	1.13- 2.26	0.007*			

*Significance level at α=0.05

## Discussion

Members of the hill tribe elderly population are living with a high burden of T2DM and HT in Thailand. There are several factors associated with HT and T2DM, such as behaviors related to daily living, culture and food practices. Most members of the hill tribe elderly population have no education and low economic status. Very few have Thai ID cards, which is usually used to access all public services in Thailand, including health care services [17]. Only one-fourth of the participants were able to speak and understand Thai, and a few people could read and write in Thai. The prevalence of T2DM and HT was 16.8% and 45.5%, respectively, of which 7.7% and 21.9% represented the incident rates for T2DM and HT, respectively. Moreover, 9.3% of T2DM participants and 37.9% of HT participants could not control their plasma glucose and blood pressure after having daily medication. The comorbidity rate was approximately one-fourth of the participants who used alcohol and smoked. The participants had a high frequency of consumption of dietary salt (64.4%), greasy food (62.5%), sweet food (50.7%) and uncooked food (44.9%). Five factors were found to be significantly associated with T2DM: tribe, exercise, BMI, parental history of T2DM, and triglycerides. Another four factors were found to be significantly associated with HT: sex, dietary salt intake, BMI, and parental history of HT.

The results of our study revealed very interesting information on the prevalence of T2DM among the hill tribe elderly populations in Thailand at 16.8%, which is 1.75 times higher than that of the Thai population [11]. We also found significant differences in prevalence among the various tribes. Meanwhile, the prevalence of HT was 45.5%, which is almost 1.6 times greater than that of the general Thai elderly population [13]. Among the participants with HT in the hill tribe elderly population, 21.9% did not know that they had HT. In taking a closer look into tribal differences, more than half of the Yao and Lisu participants had HT. This phenomenon could be attributed to the differences in culture and lifestyle among the hill tribe people, who consume alcohol and foods that are highly sweetened and salty and do not exercise regularly.

In our study, the comorbidity rate of T2DM and HT is higher than that in an Indian sample in a study of Jaya et al. [25]. However, the T2DM prevalence of our study sample is similar to that of a sample from a study conducted by Mohamed et al. [26] among the ethnic groups in northern Sudan, with a T2DM prevalence of 18.7%. Dhiraj et al. [27] reported that in different tribes of the population, there were different burdens of T2DM in the sub-Himalayan region of India. This information supports the finding that the hill tribe people in Thailand originate from Tibet [14, 16], which is close to those living in the sub-Himalayan region of India. Therefore, the T2DM and HT prevalence among the 6 hill tribes in Thailand are possibly different.

A study using a mass database in Korea reported that regular and frequent exercise led to reduced T2DM mortality and morbidly rates, particularly in the elderly population [28]. A study in Saudi Arabia also reported that sufficient physical exercise was a protective factor against T2DM development [29]. This result is similar to the finding of our study that regular exercise and highly active physical work serve as protective factors against T2DM among the hill tribe elderly populations in Thailand. Regarding BMI, Kulaya et al. [30] reported that increasing BMI was identified as a major risk factor for T2DM in the Thai population. In a study of Asian Americans in the United States, a BMI<  23 or overweight was detected as a risk factor for T2DM development [31]. Moreover, a case-control study aimed at assessing the association between BMI and T2DM in the Mid-Atlantic region found a heavy association between increasing BMI and T2DM, after controlling for all confounding factors [32]. However, in a study among Afro-Trinidadians in the United States in 2016, no significant difference in BMI was found between those who had T2DM and those who did not [33]. In our study, it was found that increasing BMI or overweight was a risk factor for T2DM in the hill tribe elderly populations.

Many studies [34–36] have reported that having a parental or family history of diabetes or first-degree relatives with diabetes was associated with the development of T2DM, which is consistent with the findings of our study. Triglyceride levels are another factor related to the development of T2DM. A retrospective longitudinal large-scale study conducted between the year 2000 and 2012 found that every 10 mg/dL increase in triglyceride levels significantly increased the risk of T2DM by 4.0% in the United States [37]. In addition, Ming et al. [38] reported that an increase in triglycerides was a risk factor for type 2 diabetes among those living in rural China. These studies present findings similar to those of this study, such that higher triglyceride levels are a risk factor for T2DM. Different tribes or races also have significant associations with T2DM. The studies of Vitor [39] and Diego et al. [40], which were conducted in the United States using different study designs, revealed that differences in the races of parents had an impact on the development of HT in their children. However, in our study, there was no significant difference in HT prevalence among the tribes.

Jugal et al. [41] reported that there were several factors associated with HT among those living in rural Delhi, India, such as older age, alcohol use, education and cholesterol levels. However, sex was not found to be associated with HT. On the other hand, Saswata et al. [42] reported that females had a greater chance of developing HT than males in a study conducted in western India. Daily food consumption is one of the predictors for HT. Daily consumption of salty foods is one of the risk factors of HT. This finding is supported by several studies [43–45] that show that dietary salt intake was highly associated with HT development in developing and developed countries and in urban and rural areas. In this study, we also found that dietary salt intake among the hill tribe elderly populations was a significant risk factor for HT development. Another factor related to HT is BMI. Alicja et al. [46] reported that both men and women had an increased risk of HT with increasing BMI, particularly among the elderly populations. A rural Chinese cohort study in 2016 [47] and a study in Bangladesh in 2017 [48] confirmed that the increase in BMI had a significant association with HT development. These findings coincide with those of our study, which revealed that an increase in BMI was associated with a greater odds of HT development among the hill tribe elderly populations in Thailand.

The study of Ghada et al. [49] in Egypt showed a strong association between a family history of HT and the development of HT in one’s offspring. A family history has been detected as a risk factor for HT among young adults and the elderly population in several countries [50–52].

Some limitations have been identified in this study, such as misunderstanding the NPO techniques before drawing blood specimens, language, and the inability to draw blood specimens in some people. Since some targeted hill tribe villages are located far away from the city, traveling to the study setting very early in the day to collect blood specimens was sometimes not practical. Other limitations included unclear information on the research procedure and not drinking and eating food for at least 8 hours before having blood drawn. Sometimes there was no cooperation from the participants, which may have occurred because they clearly did not understand the importance of laboratory interpretations. Moreover, most hill tribe elderly adults are not educated. This finding coincides with those of studies by Apidechkul et al. [53] and Apidechul [54], who reported that a high proportion of the Akha elderly population and the Lahu people were in the illiterate group. This finding could explain participants’ limited understanding of the research information and lack of cooperation with the procedure.

The researchers could not draw blood from a few participants (1.26%) because of their individual peripheral vein characteristics. However, nobody refused to provide information and a specimen. Because this lack of data would affect the predictive statistical model (logistic regressions), these participants were excluded from the analysis to ensure the accuracy of the results. Furthermore, some participants had been diagnosed as T2DM and HT before starting the study, which could possibly impact the findings of the study, particularly their knowledge, attitudes and practices, which are common limitations of the cross-sectional study design. Concerning this point, knowledge of and attitudes toward DM and HT were not included in the prediction model. Moreover, if we look closely, only attitude is significantly different among the tribes. Additionally, the number of Lahu (excess of 3participants) and Hmong (excess of 10 participants) participants exceeded the minimum requirement for the sample size due to miscommunication between the researcher and community headman. However, these excess data did not impact the results of study but rather supported the power of the tests.

Conducting research with the hill tribe people, particularly among the elderly population, required researchers to be clearly knowledgeable about the condition before reaching them. Additionally, having research assistants who were fluent in both Thai and the local hill tribe languages was an advantage for obtaining information.

## Conclusions

The hill tribe elderly populations in Thailand are living with a high burden of T2DM and HT. T2DM and HT screening programs in these populations should be implemented regularly to detect early-stage and new cases. There is an urgent need to develop proper health behavior change models to reduce BMI and the consumption of dietary salt and greasy foods among the elderly populations. Moreover, a program to encourage physical exercise is also necessary. Otherwise, Thailand must budget large amounts of money to provide care and treatment for these populations in the near future.

## Additional file

Additional file 1: Hill tirb Elderly Data. (XLSX 545 kb)

## Abbreviations

BMI: Body mass index
DALYS: Disability adjusted life year
HT: Hypertension
ID: Identification
IOC: Item objective congruence
NPO: Nothing per oral
T2DM: Type 2 diabetes mellitus
WHO: World Health Organization
